# Hemoglobin levels and transfusions in neurocritically ill patients: a systematic review of comparative studies

**DOI:** 10.1186/cc11293

**Published:** 2012-04-02

**Authors:** Philippe Desjardins, Alexis F Turgeon, Marie-Hélène Tremblay, François Lauzier, Ryan Zarychanski, Amélie Boutin, Lynne Moore, Lauralyn A McIntyre, Shane W English, Andrea Rigamonti, Jacques Lacroix, Dean A Fergusson

**Affiliations:** 1 Department of Anesthesiology, Division of Critical Care Medicine, Faculty of Medicine, Université Laval, 1050, Avenue de la Médecine, Pavillon Ferdinand-Vandry, Québec, QC, G1V 0A6, Canada; 2 Centre de Recherche FRQ-S du Centre hospitalier affilié universitaire de Québec (CHA), Hôpital de l'Enfant-Jésus, (CHA-Research Center, Enfant-Jésus Hospital), Traumatologie - Urgence - Soins Intensifs (Trauma - Emergency - Critical Care Medicine), Université Laval, 1401, 18e rue, Québec, QC, G1J 1Z4, Canada; 3 Department of Medicine, Faculty of Medicine, Université Laval, 1050, Avenue de la Médecine, Pavillon Ferdinand-Vandry, Québec, QC, G1V 0A6, Canada; 4 Department of Internal Medicine, Sections of Critical Care Medicine, of Haematology and of Medical Oncology, Faculty of Medicine, University of Manitoba, Room GC425, Health Sciences Centre, 820 Sherbrook Street, Winnipeg, MB, R3T 2N2, Canada; 5 Department of Social and Preventive Medicine, Faculty of Medicine, Université Laval, 1050, Avenue de la Médecine, Pavillon Ferdinand-Vandry, Québec, QC, G1V 0A6, Canada; 6 Department of Medicine, Division of Critical Care, Faculty of Medicine, University of Ottawa, 501 Smyth Road, Ottawa, ON, K1H 8L6, Canada; 7 Clinical Epidemiology Unit, Ottawa Hospital Research Institute, 501 Smyth Road, Ottawa, ON, K1H 8L6, Canada; 8 Departments of Anaesthesia and Critical Care Medicine, Faculty of Medicine, University of Toronto, 30 Bond Street, Toronto, ON, M5B 1W8, Canada; 9 Department of Pediatrics, Critical Care Medicine, Faculty of Medicine, Université de Montréal, 3175, Chemin Côte Sainte-Catherine, Montréal, QC, H3T 1C5, Canada

## Abstract

**Introduction:**

Accumulating evidence suggests that, in critically ill patients, a lower hemoglobin transfusion threshold is safe. However, the optimal hemoglobin level and associated transfusion threshold remain unknown in neurocritically ill patients.

**Methods:**

We conducted a systematic review of comparative studies (randomized and nonrandomized) to evaluate the effect of hemoglobin levels on mortality, neurologic function, intensive care unit (ICU) and hospital length of stay, duration of mechanical ventilation, and multiple organ failure in adult and pediatric neurocritically ill patients. We searched MEDLINE, The Cochrane Central Register of Controlled Trials, Embase, Web of Knowledge, and Google Scholar. Studies focusing on any neurocritical care conditions were included. Data are presented by using odds ratios for dichotomous outcomes and mean differences for continuous outcomes.

**Results:**

Among 4,310 retrieved records, six studies met inclusion criteria (*n *= 537). Four studies were conducted in traumatic brain injury (TBI), one in subarachnoid hemorrhage (SAH), and one in a mixed population of neurocritically ill patients. The minimal hemoglobin levels or transfusion thresholds ranged from 7 to 10 g/dl in the lower-Hb groups and from 9.3 to 11.5 g/dl in the higher-Hb groups. Three studies had a low risk of bias, and three had a high risk of bias. No effect was observed on mortality, duration of mechanical ventilation, or multiple organ failure. In studies reporting on length of stay (*n *= 4), one reported a significant shorter ICU stay (mean, -11.4 days (95% confidence interval, -16.1 to -6.7)), and one, a shorter hospital stay (mean, -5.7 days (-10.3 to -1.1)) in the lower-Hb groups, whereas the other two found no significant association.

**Conclusions:**

We found insufficient evidence to confirm or refute a difference in effect between lower- and higher-Hb groups in neurocritically ill patients. Considering the lack of evidence regarding long-term neurologic functional outcomes and the high risk of bias of half the studies, no recommendation can be made regarding which hemoglobin level to target and which associated transfusion strategy (restrictive or liberal) to favor in neurocritically ill patients.

## Introduction

Anemia is highly prevalent in the intensive care unit (ICU), with up to 95% of critically ill patients developing subnormal hemoglobin levels by day 3 [1]. Likewise, 20% to 53% of patients receive red blood cell (RBC) transfusions to correct anemia during their ICU stays [2]. However, allogenic RBC transfusions carry risks that may adversely affect clinical outcomes [3,4]. Evidence suggests that it is safe to adopt a lower transfusion threshold for the general medical/surgical ICU population [1,4-8]. This has led to a paradigm shift concerning RBC transfusions in the ICU, with most guidelines now recommending hemoglobin levels around 7 g/dl for transfusion in patients without significant comorbidities to minimize exposure to allogenic blood [9-11].

Most studies of transfusion thresholds have focused on a general medical/surgical ICU population but not on specific, and potentially more vulnerable, subpopulations of critically ill patients, such as those with acute neurologic conditions [12]. Indeed, neurocritically ill patients may represent an exception to the rationale for using low transfusion triggers because impaired oxygen delivery is a crucial modifiable factor in brain ischemia and secondary brain injury [13,14]. The optimal hemoglobin level for cerebral oxygen delivery in these patients is still unknown [15]. Moreover, data on which clinicians have to rely in decision making is discordant, as both anemia and RBC transfusion have been observed to be associated with unfavorable clinical outcomes in neurocritically ill patients [16-18].

Current guidelines for the optimal transfusion threshold in neurocritical care populations are scarce, and their recommendations are conflicting about which threshold to favor [19,20]. Several narrative studies have aimed to summarize the topic [15-18], but no systematic review has been designed to address specifically the question of transfusion thresholds in the neurocritical population. We thus undertook a systematic review of comparative studies to evaluate the effects of hemoglobin levels and RBC transfusion strategies on clinical outcomes in adult and pediatric neurocritically ill patients.

## Materials and methods

This systematic review was designed in accordance with the PRISMA statement for systematic reviews and meta-analyses [21]. A study protocol was developed and followed through every step of the review.

### Search strategy

We designed a search strategy for Ovid MEDLINE (1949 to the present), the Cochrane Central Register of Controlled Trials (1974 to Issue 1, 2011), as well as Embase and Embase Classic (1974 to the present). Abstracts and conference proceedings were searched in BIOSIS previews (1926 to the present) and Web of Science (1898 to the present), whereas the grey literature was searched by using Google Scholar. We sought both randomized controlled trials (RCTs) and comparative nonrandomized studies, both prospective or retrospective. No restriction based on language, year, or type of publication was applied. Keywords and Medical Subject Headings (MeSH) terms (or their EMTREE equivalents) pertaining to the population (neurocritical care) and to the exposure (hemoglobin levels, RBC transfusion, anemia) were combined to form the search strategy (Additional file 1). We used clinicaltrials.gov, controlled-trials.com, and strokecenter.org websites to identify unpublished and ongoing studies. Reference lists from relevant reviews and included articles were manually searched to identify missed studies. The last iteration of the search process was completed on January 31, 2011.

### Selection of studies

We included comparative studies evaluating the effect of hemoglobin levels on clinical outcomes of neurocritically ill patients admitted to an ICU. Studies were included if at least two different hemoglobin thresholds, levels, targets, or RBC transfusion strategies were compared. Neurocritical conditions encompassed but were not limited to subarachnoid hemorrhage (SAH), stroke, traumatic brain injury (TBI), intracerebral hemorrhage (ICH), and any cerebral neurosurgical conditions. Studies on sickle cell anemia and scoliosis surgery were excluded. We also excluded studies in neonates (< 28 days), but all other age groups were considered.

Two independent reviewers (PD, MHT) screened the studies identified from the systematic search. Non-English language articles were translated as required. A Cohen kappa statistic was calculated to quantify the interrater agreement concerning inclusion of studies. In case of discrepancy, a third reviewer (AFT) was involved to settle the disagreement. Search results from Web of Science, from grey literature sources, and from reference lists of identified studies were reviewed and adjudicated by a single reviewer (PD).

### Data-collection process

A standardized abstraction form was developed and tested before data collection. Data abstraction was conducted independently, and in duplicate, by two reviewers (PD, MHT). When judged necessary, missing information was requested from corresponding authors.

The primary outcome measure was all-cause mortality at any given time point. Secondary outcomes were neurologic status (irrespective of the scale used), ICU length of stay, hospital length of stay, duration of mechanical ventilation, surrogate measures of brain oxygen delivery, complications (including vasospasm and multiple organ dysfunction score) [22], and serious adverse events (thromboembolic events, myocardial infarction, pulmonary edema or volume overload, transfusion-related acute lung injury (TRALI), and infection). Data pertaining to the study design were also retrieved, as well as characteristics of patients that could act as confounders and affect the outcomes of interest, including age, sex, disease severity, comorbidities, incidence of hypoxemia, incidence of hypotension, and baseline hemoglobin. Information on blood transfusion and the nature, timing, and frequency of co-interventions (hemodilution, blood-conservation strategies, erythropoietin analogues, and use of other blood products) were recorded.

### Assessment of methodologic quality and risk of bias

Two reviewers (PD, MHT) independently evaluated the risk of bias in included studies. We used the Cochrane Collaboration tool for assessing risk of bias in RCTs, which was customized for the focus of the review [23]. We judged the overall risk of bias of individual studies as low, moderate, high, or unclear [23]. Additionally, we used the Downs and Black checklist [24] to assess the methodologic quality of both RCTs and nonrandomized studies. This checklist has been validated for reliability and external validity. We put emphasis on how study authors dealt with confounding factors mentioned earlier in nonrandomized studies. The last item of the Downs and Black checklist is an assessment of the adequacy of the sample size of the study, which we performed assuming a two-sided *P *value of 0.05, 80% power, and a 10% relative difference for the main outcome measure.

### Statistical analysis

A meta-analytic approach was planned by using Mantel-Haenztel random-effect models, if deemed appropriate. We presented outcome data by using odds ratios with 95% confidence intervals. An odds ratio of less than 1 suggests a lower rate of the event among the patients exposed to lower hemoglobin levels. Continuous data, such as length of stay and physiologic parameters, were reported as mean or median. We summarized continuous data as mean difference with 95% confidence intervals. We converted hematocrit to hemoglobin by using a standard published equation [25] (Hb [g/dl] = Hct [%]/3). All data were compiled in Review Manager (version 5.0; The Cochrane Collaboration). *A priori *sensitivity analyses were planned to explore heterogeneity in study findings, based on age, type of neurocritical condition, risk of bias, and presence of co-interventions.

## Results

### Search results

**Figure 1:**
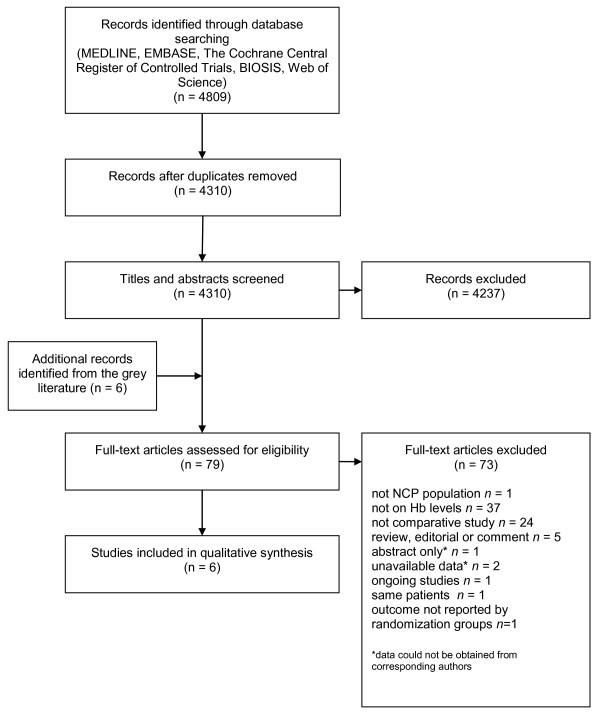
**Flow diagram of study selection**. Hb, hemoglobin concentration; NCP, neurocritically ill patient.

Our literature search yielded 4,310 studies from major databases after removal of duplicate records (Figure 1). Seven studies were deemed potentially elligible [6,26-31], but one was excluded, as it reported only summary data for the overall group [31]. The authors of the latter study were contacted and confirmed that data from each study group were unavailable. Therefore, six studies were included (number of patients = 537) [6,26-30]. The overall interrater agreement between reviewers on inclusion was high (Cohen kappa = 0.80). Discrepancies were resolved with the input of a third reviewer on two occasions.

Data for the subgroup of neurocritically ill patients from a previous RCT on transfusion thresholds in pediatric ICUs were obtained from the authors for one study [6]. Attempts to contact the authors of two other studies to obtain data on subgroups of published RCTs were unsuccessful [32,33]. Two abstracts of potentially relevant studies were retrieved through the review of conference proceedings; however, one reported data on a study already included in our review [29,34], whereas the other abstract [35] had not yet been published as a full report. Additional data for this study could not be obtained from the corresponding author. Of note, one relevant ongoing study was identified [36].

### Study characteristics

Among the six studies included, three were from the United States [28-30], two from Canada [26,28], and one was completed in Switzerland [27] (Table 1). All studies were published in peer-reviewed English language journals within the last 5 years, and the period over which the studies were conducted spanned from 1994 to 2011. Only one study involved pediatric patients [6], whereas the other five studies took place in adult ICUs. One study was an RCT [30], one was a subgroup analysis of an RCT in the overall critically ill adult population (Transfusion Requirements in Critical Care (TRICC) trial) [26], one was an unpublished subgroup analysis of an RCT in the critically ill pediatric population (Transfusion Requirements in the Pediatric Intensive Care Unit (TRIPICU) trial) [6], and three were retrospective cohort studies [27-29]. The main diagnosis of patients was traumatic brain injury (*n *= 4) [26-29] and subarachnoid hemorrhage (*n *= 1) [30]. The subset data used from the TRIPICU study [6] included patients representing different neurocritical care conditions: TBI (*n *= 36), ICH (*n *= 11), brain tumors (*n *= 3), neurosurgery (excluding scoliosis surgery) (*n *= 9), cerebral edema (*n *= 5) and other space-occupying injuries (*n *= 2).

**Table 1:** Description of included studies

Study	Design	Number	Setting	Population	Outcomes measured	Confounding factors considered in the analyses	Follow-up period
McIntyre *et al*. (2006)	Subgroup of a previously published RCT	67	25 adult ICUs	Moderate to severe TBI	Mortality, MODS, ICULOS, HLOS, RBCT during ICU, infection, physician nonadherence	Age, APACHE score, use of pulmonary artery catheter, mechanical ventilation, vasopressor agents	60 days
Flückiger *et al*. (2010)	Retrospective cohort study	139	One adult ICU	Severe TBI	In-hospital mortality, ICU complications, GOSe	Worst Hct during ER/OR phase, transfusion and volume management during ER/OR phase, complications and transfusions during ICU phase	6 months
Lacroix *et al*. (2007)	Subgroup of a previously published RCT	66	19 pediatric ICUs	TBI ICH Elective neurosurgery Other space-occupying injuries	MODS, progression of MODS, ICULOS, duration of mechanical ventilation, mortality, infections, transfusion reaction, adverse events	Age, country, severity of illness, anemia, admission diagnosis	28 days
George *et al*. (2008)	Retrospective cohort study	82	Two adult ICUs	Severe TBI	Mortality, pneumonia, UTI, bacteremia, sepsis, decubitus ulcer, myocardial infarction, seizure, DVT, pulmonary embolus	Age, gender, motor and total GCS, admission BAL, head and neck AIS score, ISS, presence of SAH, min. ICU Na and Hb levels, RBCT, any complication	NR
Warner *et al*. (2010)	Retrospective cohort study	139	One adult ICU	Moderate to severe TBI	GOSe, FSE, mortality	Age, head AIS score, days with Hb < 10 g/dl, RBCT performed, volume of RBCT, highest serum glucose, days with serum glucose > 200 mg/dl, HLOS, admission GCS, mild versus severe TBI, reason and timing for RBCT	6 months
Naidech *et al*. (2010)	Randomized controlled trial	44	One adult ICU	SAH at high risk of vasospasm, cerebral infarction	Fever, ventilator-free days, vasospasm, pulmonary edema or respiratory distress NIH Stroke Scale, modified Rankin Scale	Age, WFNS score on admission, history of hypertension or diabetes	3 months

AIS, abbreviated injury scale; APACHE, Acute Physiology and Chronic Health Evaluation; BAL, blood alcohol level; DVT, deep vein thrombosis; ER, emergency room; FSE, functional status examination; GCS, Glasgow Coma Scale; GOSe, Extended Glasgow Outcome Scale; Hb, hemoglobin concentration; Hct, hematocrit; HLOS, hospital length of stay; ICH, intracerebral hemorrhage; ICU, intensive care unit; ICULOS, intensive care unit length of stay; ISS, injury severity score; MODS, multiple organ dysfunction syndrome; NIH, National Institutes of Health; NR, not reported; OR, operating room; RBCT, red blood cell transfusion; RCT, randomized controlled trial; SAH, subarachnoid hemorrhage; TBI, traumatic brain injury; UTI, urinary tract infection; WFNS, World Federation of Neurosurgeons.

All six studies compared two groups of patients whose targets or observed hemoglobin levels differed, having or having not received an RBC transfusion (Table 2). Three studies, all of which were RCTs, described mandatory transfusion protocols [6,26,30] based on specified hemoglobin thresholds. Two observational studies [28,29] compared groups of patients who received or did not receive RBC transfusion for a specified baseline hemoglobin range. One cohort study compared patients who reached and did not reach a target hemoglobin level after the resuscitation phase of TBI [27].

**Table 2:** Description of red blood cells transfusion strategies or hemoglobin levels compared in included studies

			Severity of the disease						Exposure				
						
Study	Groups	**Age (years)**^**a**^	**GCS**^**a**^	*P *value	**ISS**^**a**^	*P *value	Mean baseline Hb (g/dl)	*P *value	Mandatory RBCT protocol	RBCT strategy	Patients transfused (%)	**Hb achieved (g/dl)**^**a**^	*P *value
McIntyre *et al*. (2006)	Liberal (*n *= 38)	39.8 ± 18.1	7.5 ± 3.6	NR	31.3 ± 13.0	NR	NR		Yes	Threshold 10 g/dl Target range 10-12 g/dl	38 (100)	10.5 ± 0.6	< 0.0001
	Restrictive (*n *= 29)	41.7 ± 20.4	7.3 ± 3.4		29.8 ± 14.0					Threshold 7 g/dl Target range 7-9 g/dl	17 (59)	8.5 ± 0.7	

Flückiger *et al*. (2006)	Higher Hb (*n *= 102)	NR	NR		28.65	NR	12.4	NR	No	Hb ≥9.3 g/dl by the end of OR phase	31 (30)	11.1	NR
	Lower Hb (*n *= 37)				32.70		10.4			Hb < 9.3 g/dl by the end of OR phase	32 (86)	8.4	

Lacroix *et al*. (2007)	Liberal (*n *= 36)	5.5 ± 5.1	NR		NR		8.2 ± 0.9	NR	Yes	Threshold 9.5 g/dl Target range 11-12 g/dl	35 (97)	10.6 ± 1.0	< 0.0001
	Restrictive (*n *= 30)	5.2 ± 4.7					7.7 ± 1.0			Threshold 7 g/dl Target range 8.5-9.5 g/dl	20 (67)	8.9 ± 1.0	

George *et al*. (2008)	Higher Hb (*n *= 43)	54.6 ± 23.9	4.2 ± 1.7	0.013	25.0 ± 3.3	0.12	NR		No	Received at least 1 RBCT when Hb was between 8 and 10 g/dl	43 (100)	NR	
	Lower Hb (*n *= 39)	52.6 ± 19.5	5.5 ± 2.4		23.5 ± 4.5					Did not receive RBCT when Hb was between 8 and 10 g/dl	0 (0)		

Warner *et al*. (2010)	Higher Hb (*n *= 76)	39.8 ± 19.3	7.1 ± 5.0	0.007	29.8 ± 10.7	0.085	30.0 ± 3.9	0.663	No	Received at least 1 RBCT when Hb was between 7 and 10 g/dl	76 (100)	NR	
	Lower Hb (*n *= 63)	40.9 ± 20.6	9.7 ± 5.1		26.5 ± 9.1		29.8 ± 6.8			Did not receive RBCT when Hb was between 7 and 10 g/dl	0 (0)		

Naidech *et al*. (2010)	Higher Hb (*n *= 21)	54.1 ± 14.9	NR		NR		13.4	NR	Yes	Threshold 11.5 g/dl	20 (95)	12.3 ± 0.3	< 0.0001
	Lower Hb (*n *= 23)	59.2 ± 11.9					13.1			Threshold 10 g/dl	19 (82)	11.1 ± 0.4	

GCS Glasgow Coma Scale; Hb hemoglobin concentration; ISS injury severity score; NR not reported; OR operating room; RBCT red blood cell transfusion. **^a^**Mean ± standard deviation.

Among studies, lower hemoglobin levels or thresholds ranged from 7 g/dl to 10 g/dl, whereas higher hemoglobin levels or thresholds ranged from 9.3 to 11.5 g/dl. One study divided patients on the basis of achieved hemoglobin levels above or below 9.3 g/dl [27]. The duration of exposure to these hemoglobin levels varied among studies. In three of six studies [6,26,30], hemoglobin levels were maintained during the entire ICU stay. In the remaining three studies [27-29], hemoglobin levels were measured at only one point in time, and thus the exposure was not necessarily sustained. Among the four studies [6,26,27,30] for which the mean hemoglobin levels during the period of exposure could be obtained, all observed a statistically significant difference of at least 1 g/dl (range, 1.2 to 2.78 g/dl) between the two comparison groups. The follow-up period varied between 28 days and 6 months among studies.

One study reported the use of triple-H (hemodilution, hypertension, hypervolemia) therapy in patients with symptomatic vasospasm afer a subarachnoid hemorrhage [30]. The incidence of vasospasm was not significantly different in the higher-hemoglobin and lower-hemoglobin groups (24% and 22%, respectively; *P *= 1.00). The use of fresh-frozen plasma was reported in two studies [6,28]. In one study, only two patients (one in each group) received fresh-frozen plasma [6]; whereas in the other, a mean of one more unit was given in the higher-Hb group (*P *= 0.046) [28]. No other relevant co-interventions susceptible to interfere with hemoglobin levels and outcomes were documented.

### Assessment of methodologic quality and risk of bias

All RCTs and subgroups of RCTs presented an overall low risk of bias (Table 3) [6,26,30]. Given the nature of the intervention, no study was blinded to allocation during ICU management. All studies reported adequate allocation concealment before and during enrollment. Data analysis of these three studies respected the intention-to-treat principle. Nonrandomized studies generally were of lower methodologic quality than RCTs and subgroup analyses of RCTs. Two of the six studies [27,30] did not adjust study estimates for important confounding factors, such as severity of the baseline condition.

**Table 3:** Risk of bias and methodologic quality assessment of included studies

Study	Rating based on Cochrane risk-of-bias tool							Downs and Black checklist					
	
										Internal validity			
												
	Sequence generation	Allocation concealment	Blinding	Incomplete outcome data	Selective outcome reporting	Other bias	Summary	Reporting	External validity	Bias	Confounding	Power	Total
								/11	/3	/7	/6	/5	/32
RCTs or subgroup of RCTs													
												
McIntyre *et al*. 2006^**a**^	Low	Low	High	Low	Low	Low	Low	10	3	6	5	0	24
Lacroix *et al*. 2007^**a**^	Low	Low	High	Low	Low	Low	Low	11	3	5	6	0	25
Naidech *et al*. 2010	Low	Low	Unclear	Low	Low	Low	Low	7	1	6	5	2	21
Nonrandomized studies													
												
Flückiger *et al*. 2010								5	3	6	4	0	18
George *et al*. 2008								9	2	4	3	0	18
Warner *et al*. 2010								9	2	6	3	1	21

RCT randomized controlled trial. **^a^**Cochrane risk-of-bias tool applied on initial RCT.

### Outcome measures

Given the substantial heterogeneity observed in study designs and participants of included studies, a formal meta-analysis was considered to be inappropriate, and data were not pooled. Study data were therefore descriptively synthesized. For the same reason, we did not quantify statistical heterogeneity, and we did not conduct sensitivity analyses as planned.

### Mortality

Five studies presented data on mortality [6,26-29]. None showed a statistically significant effect of lower Hb levels when compared with higher Hb levels (Table 4).

**Table 4:** Effects of lower versus higher hemoglobin levels on mortality

		No. of events/No. of participants		
			
Study	Time frame	Lower Hb	Higher Hb	Odds ratio (95% CI)
McIntyre *et al*. 2006	30 days	5/29	5/38	1.38 [0.36, 5.29]
Flückiger *et al*. 2010	In-hospital	17/37	34/102	1.70 [0.79, 3.66]
Lacroix *et al*. 2007	28 days	2/30	1/36	2.50 [0.22, 29.01]
George *et al*. 2008	In-hospital	11/39	15/43	0.73 [0.29, 1.87]
Warner *et al*. 2010	6 months	6/63	13/76	0.51 [0.18, 1.43]

CI, confidence interval; Hb, hemoglobin concentration.

### Neurologic outcomes

One study reported short and mid-term neurologic outcomes, as defined by the National Institutes of Health (NIH) Stroke Scale (14 days) and the modified Rankin scale (14 and 28 days, and 3 months) [30], and one study evaluated long-term (≥6 months) functional neurologic outcome by using the extended Glasgow outcome scale (GOSe) [29]. When looking at the former, the median scores on the NIH Stroke scale were 1 (Q1 to Q3: 0 to 9.75) in the higher-Hb group versus 2 (0 to 16) in the lower-Hb group (*P *> 0.10). At 14 days, 13 patients in the higher-Hb group were considered "independent" on the modified Rankin Scale as opposed to 10 in the lower-Hb group, but the difference was not statistically significant (*P *= 0.25). Findings at 28 days (14 versus 16 patients; *P *= 0.34) and at three months (18 versus 20 patients; *P *= 1.00) were similar and did not achieve statistical significance [30]. Compared with a higher-Hb target group (patients transfused when Hb was between 7 and 10 g/dl), investigators of the second study observed a statistically significant increase in the GOSe score (5.7 versus 3.9; *P *< 0.0005, higher score meaning better outcome) at six months in patients in the lower Hb target group (not transfused when Hb was between 7 and 10 g/dl). This difference remained significant after adjustment was made for the Glasgow Coma Scale score on admission (5.4 ± 0.3 versus 4.1 ± 0.2; *P *= 0.0005) [29].

### Duration of mechanical ventilation

Two studies [6,30] reported the duration of mechanical ventilation and observed no significant difference in the number of days on mechanical ventilation between the higher and lower hemoglobin-level groups (mean difference, 0.57 days (95% CI, -1.78 to 2.92) and -0.63 days (95% CI, -1.85 to 0.60), respectively).

### Length of stay

Four of the six studies [6,26,28,29] reported length of stay (Table 5). A significantly different mean ICU length of stay in favor of the lower-Hb group (11.0 days) versus the higher-Hb group (16.7 days; *P *= 0.02) was observed in one study [28]. In this study, a similar effect was observed for hospital stay, but it did not reach statistical significance. A significant difference in mean hospital stay was, however, observed in one study (mean difference, -11.40 (95% CI, -16.0 to -6.70)) in favor of the lower-Hb group [29]. In contrast, results of two other studies did not show a significant difference in ICU length of stay between the two study groups [6,26].

**Table 5:** Effects of lower versus higher hemoglobin levels on length of stay

Study	Time frame	Measure	Lower Hb	Higher Hb	*P *value	Mean difference (95% CI)
McIntyre *et al*. 2006	ICU	Median (IQ range)	10 (5-21)	8 (5-11)	0.26	Not estimable
	Hospital		27 (14-39)	30.5 (17-47)	0.72	
Lacroix *et al*. 2007	ICU	Mean (± SD)	8.0 ± 5.2	9.9 ± 7.0	0.37	-1.9 [-4.9, 1.1]
George *et al*. 2008	ICU	Mean (± SD)	11.0 ± 8.6	16.7 ± 12.2	0.02	-5.7 [-10.3, -1.1]
	Hospital		13.0 ± 9.9	17.7 ± 11.7	0.09	-4.7 [-9.4, 0.02]
Warner *et al*. 2010	Hospital	Mean (± SD)	11.7 ± 7.4	23.1 ± 17.8	< 0.0005	-11.4 [-16.1, -6.7]

CI, confidence interval; Hb, hemoglobin concentration; ICU, intensive care unit; IQ, interquartile; SD, standard deviation.

### Organ failure

Two studies addressed organ failure by using the multiple organ dysfunction score (MODS) [6,26]. In both studies, neither the progression of organ failure nor the emergence of a new MODS was significantly different between the restrictive and the liberal groups. In the subgroup of neurocritically ill patients from the TRIPICU study, the proportion of patients with new or worsening MODS was 16.6% in the restrictive group versus 8.3% in the liberal group, but this difference did not achieve statistical significance (*P *= 0.45) [6]. In the subgroup analysis of patients with traumatic brain injury from the TRICC trial, the worsening of MODS was also similar in both intervention arms (3.4 restrictive versus 4.5 liberal; *P *= 0.49) [26].

### Serious adverse events

We assessed for the reporting of myocardial infarction, pulmonary edema and volume overload, transfusion-related acute lung injury, thromboembolic events, and infections (Table 6). At least one serious adverse event was included as a secondary outcome in five studies [6,26-28,30], but no study reported a systematic method to screen for serious adverse events. The reported incidence of adverse events is shown in Table 6.

**Table 6:** Reported adverse events

Study	Number of patients with adverse events									
	**Myocardial infarction**		**Pulmonary edema/volume overload**		**TRALI**		**Thromboembolism**		**Infections**	
	
	**Lower Hb**	**Higher Hb**	**Lower Hb**	**Higher Hb**	**Lower Hb**	**Higher Hb**	**Lower Hb**	**Higher Hb**	**Lower Hb**	**Higher Hb**

McIntyre *et al*.	NR	NR	NR	NR	NR	NR	NR	NR	2	2
Flückiger *et al*.	NR	NR	NR	NR	NR	NR	NR	NR	NR	NR
Lacroix *et al*.	NR	NR	NR	NR	NR	NR	NR	NR	10	14
George *et al*.	1	0	NR	NR	NR	NR	2	10	2	1
Warner *et al*.	NR	NR	NR	NR	NR	NR	NR	NR	NR	NR
Naidech *et al*.	NR	NR	8	3	NR	NR	NR	NR	2	2

Hb, hemoglobin concentration; NR, not reported; TRALI, transfusion-related acute lung injury.

## Discussion

In this systematic review, despite our thorough search of the literature, we identified very few comparative studies of transfusion strategies conducted in different pediatric or adult neurocritically ill populations. Insufficient data exist to refute or confirm a mortality benefit associated with the maintenance of lower or higher hemoglobin level nor to support a consistent effect on organ failure and duration of mechanical ventilation. A potential decrease in hospital and ICU length of stay was observed, but only in studies with high risk of bias. Interestingly, only two studies presented long-term functional outcomes but were not designed to evaluate a plausible clinical effect. These results underscore the paucity of evidence to justify the use of a restrictive or a liberal strategy for RBC transfusions in neurocritically ill patients.

Many theoretic effects of maintaining low hemoglobin levels in neurocritically ill patients have been described in previous experimental studies. Lower hemoglobin concentration is directly related to lower blood viscosity [37]. In mild anemia, this decrease in viscosity causes an increase in cerebral blood flow (CBF) through a direct rheologic effect and improves cerebral oxygen delivery (DO_2_) [38]. However, more-severe anemia may be detrimental in neurocritically ill patients because the decline in CaO_2 _may not be compensated by the usual CBF regulation mechanisms, which are mitigated in brain injury. On clinical grounds, anemia has repeatedly been shown to be associated with unfavorable outcomes in patients with TBI [39,40], although other studies have not confirmed this relation [41,42]. In patients with SAH, anemia has mostly been associated with unfavorable outcomes [43-45]. Recent microdialysis studies showed that cerebral metabolism becomes impaired at Hb values lower than 9 g/dl [46,47].

RBC transfusions are known to improve physiologic measures such as brain oxygen tension in a majority of patients with TBI [31,48-50]. Retrospective cohort studies in patients with SAH reported an association between the correction of anemia with RBC transfusion and unfavorable outcomes [51,52], more complications [53], and vasospasm [54]. Both anemia and RBC transfusion have thus been associated with worse clinical outcomes in different neurocritically ill patients.

Interestingly, we did not observe similar findings in our study. This is likely to be explained by the fact that we studied the impact of the exposure to Hb levels and transfusion strategies on outcomes, unlike most previous studies, which evaluated the impact of RBC transfusions (as a risk factor for a specific oucome measure rather than an intervention), regardless of the hemoglobin thresholds or Hb levels. By doing so, we aimed to avoid two potential biases. The first pertains to anemia, which often occurs in sicker patients along with confounding variables such as a greater volume of sampled blood in patients with more severe diseases [48]. Thus, it is prone to confounding despite adjustment for disease severity. The second is a potential multicolinearity bias concerning RBC transfusion and anemia. These two variables are strongly linked, given that anemic patients are predisposed to receive more RBC transfusions because of the natural tendency of physicians to give transfusions to sicker patients. To separate the respective effects of anemia and RBC transfusion, we opted to focus on differential transfusion strategies. Therefore, our approach aimed to determine a potential inflexion point (typically the mean of a hemoglobin threshold for transfusion) at which the benefits of correcting anemia surpass the detrimental effects of RBC transfusion.

One of the main limitations of our study pertains to the significant inconsistency in observed summary estimates. This may in part be due to the heterogeneity in study designs. Outcomes were assessed at various time points, and the exposure to Hb levels varied across studies. In particular, the overlaping of ranges between lower and higher Hb levels in various study groups limited direct comparison between studies. Moreover, the presence of a mandatory transfusion protocol in RCTs versus the passive observation of different hemoglobin levels in nonrandomized studies can lead to a difference in observed effects. It would be misleading to liken the data obtained from the nonrandomized studies to transfusion strategies. Accordingly, we did not pool results from RCTs and nonrandomized studies to avoid generating a more-precise but biased pooled estimate [23]. Still, we believe the comparison within each trial between groups of higher and lower Hb levels, whether by a definite transfusion trigger or by observed exposure to different Hb levels, is valid.

Some other concerns may affect our findings. The first one is obviously the scarcity of RCTs in this neurocritically ill population, despite the large number of retrospective studies in this field. The retrieved studies are mainly in the TBI population, with only two of the six studies focusing on the non-TBI population. Therefore, we cannot extrapolate our findings to stroke and ICH.

Second, most included studies were underpowered to evaluate clinically significant outcomes, making the detection of a difference in these outcomes unlikely, if present. Wide confidence intervals around estimates also stem from small sample sizes.

Third, lack of data on many relevant outcomes, such as long-term neurologic functional status or organ dysfunction, precluded the pooling of data. Even more worrying is the lack of systematic reporting of adverse events associated with RBC transfusion, because one of the main reasons to withhold RBC transfusion is to prevent, at least theoretically, these adverse events.

Finally, the methodologic quality of three of the six included studies was not optimal, although RCTs were considered to have a low risk of bias. In particular, issues with blinding and confounding cast a shadow on the robustness of findings.

## Conclusions

In our study, we could not refute or confirm a difference in mortality or long-term neurologic outcomes between RBC transfusion strategies in neurocritically ill patients. Considering the lack of evidence regarding these clinically significant outcomes and the risk of bias of studies, no recommendation can be made about which transfusion strategy to favor in neurocritically ill patients; no evidence exists that maintenance of a lower or a higher hemoglobin level is superior in this specific population. Interestingly, despite how common RBC transfusions can be in neurocritically ill patients, there is a paucity of evidence about when it is appropriate to transfuse.

Ultimately, our findings suggest that research in transfusion therapy in neurocritical care is still in its infancy. Future research on the management of anemia and RBC therapy is warranted. We believe such research should assess long-term neurologic functional status, thoroughly seek adverse events, and encompass different neurocritically ill populations, such as traumatic brain injury, subarachnoid hemorrhage, and stroke.

## Key messages

• Very few comparative studies have been conducted on the effect of restrictive versus liberal RBC-transfusion strategies in neurocritically ill patients. These studies are of variable methodologic quality, and most of them did not evaluate long-term functional outcomes.

• Insufficient evidence exists to refute or confirm a mortality benefit associated with the maintence of low (restrictive) or high (liberal) hemoglobin levels on the incidence of organ failure or on the duration of mechanical ventilation.

• We observed a potential decrease in hospital and ICU length of stay associated with lower-Hb levels exposures.

• Our systematic review underscores the paucity of evidence regarding the use of a restrictive or a liberal strategy for RBC transfusions in neurocritically ill patients.

## Abbreviations

CBF: cerebral blood flow; GOSe: Extended Glasgow Outcome Scale; Hb: hemoglobin; Hct: hematocrit; ICH: intracerebral hemorrhage; ICU: intensive care unit; MeSH: medical subject heading; MODS: multiple organ dysfunction score; NIH: National Institutes of Health; RBC: red blood cell; RCT: randomized controlled trial; SAH: subarachnoid hemorrhage; TBI: traumatic brain injury; TRALI: Transfusion-related Acute Lung Injury; TRICC: Transfusion Requirements in Critical Care; TRIPICU: Transfusion Requirements in the Pediatric Intensive Care Unit.

## Competing interests

The authors declare that they have no competing interests.

## Authors' contributions

PD, AFT, FL, RZ, LM, LM, SWE, and DAF contributed to the conception and design of the study. PD and MHT evaluated the eligibility of studies and extracted data. PD and AFT performed and reviewed the analyses. PD and AFT drafted the manuscript. All authors participated in the interpretation of the data, the critical review of the manuscript, and approved the final version.

## Authors' information

Drs Turgeon and Lauzier are recipients of a research career award from the Fonds de la Recherche Québec-Santé (FRQ-S). Dr Turgeon is supported by the Traumatology Research Consortium of the FRQ-S. Drs Moore, McIntyre, and Fergusson are recipients of New Investigator Awards from the Canadian Institutes for Health Research (CIHR). Dr Zarychanski is a recipient of an RCT mentorship award from the CIHR.

## Supplementary Material

Additional file 1 **OVID MEDLINE search strategy**. Search strategy used in MEDLINE (Ovid) using keywords and Medical Subject Headings (MeSH) terms pertaining to the population (neurocritical care) and to the exposure (hemoglobin levels, RBC transfusion, anemia). Click here for file
