# Supplementary material for: Hemoglobin levels and transfusions in neurocritically ill patients: a systematic review of comparative studies
Source: Crit Care. 2012 Apr 2;16(2):R54. doi: 10.1186/cc11293 (PMC3681381; doi:10.1186/cc11293)
Supplement: Additional file 1 — OVID MEDLINE search strategy. Search strategy used in MEDLINE (Ovid) using keywords and Medical Subject Headings (MeSH) terms pertaining to the population (neurocritical care) and to the exposure (hemoglobin levels, RBC transfusion, anemia). [file cc11293-S1.DOC]

**Additional file I**

**OVID MEDLINE search strategy**

1. blood transfusion$.mp. OR exp Blood Transfusion/
2. RBC transfusion$.mp. OR red blood cell$ transfusion$.mp.
3. packed red cell$ transfusion$.mp. OR PRC transfusion$.mp.
4. erythrocyte$ transfusion$.mp. OR exp Erythrocyte Transfusion/
5. h?emoglobin threshold.mp. OR h?emoglobin concentration.mp. OR h?emoglobin level.mp. OR h?emoglobin target.mp.
6. exp Hemoglobins/
7. h?ematocrit threshold.mp. OR h?ematocrit level.mp. OR h?ematocrit target.mp
8. exp Hematocrit/
9. transfusion threshold.mp. OR transfusion trigger.mp. OR transfusion strateg*.mp.
10. an?emia.mp. OR exp Anemia/
11. 1 OR 2 OR 3 OR 4 OR 5 OR 6 OR 7 OR 8 OR 9 OR 10
12. glycated.mp. OR glycosylated.mp. OR HbA1c.mp. OR exp Hemoglobin A, Glycosylated/
13. sickle cell.mp. OR exp Hemoglobin, Sickle/
14. 11 NOT (12 OR 13)
15. head trauma.mp. OR head injur*.mp.
16. brain trauma.mp. OR brain injur*.mp. OR exp Brain Injuries/
17. cerebral trauma.mp. OR cerebral injur*.mp.
18. craniocerebral injur*.mp. OR craniocerebral trauma.mp. OR exp Craniocerebral Trauma/
19. traumatic brain injur*.mp.
20. 15 OR 16 OR 17 OR 18 OR 19
21. subarachnoid h?emorrhage.mp. OR exp Subarachnoid Hemorrhage/
22. SAH.mp.
23. intracranial h?emorrhage.mp. OR exp Intracranial Hemorrhages/
24. 21 OR 22 OR 23
25. stroke.mp.
26. exp Stroke/
27. cerebrovascular accident$.mp.
28. cerebral infarction.mp.
29. 25 OR 26 OR 27 OR 28
30. neurocritical care.mp.
31. neurologic intensive care.mp. OR neurointensive care.mp.
32. neurotrauma.mp.
33. neurosurgical procedure$.mp. OR exp Neurosurgical procedures/
34. 30 OR 31 OR 32 OR 33
35. 20 OR 24 OR 29 OR 34
36. 14 AND 35
37. exp Animals/
38. exp Humans/
39. 37 NOT (37 AND 38)
40. 36 NOT 39
